# Implications of Three-Dimensional Cell Culture in Cancer Therapeutic Research

**DOI:** 10.3389/fonc.2022.891673

**Published:** 2022-05-12

**Authors:** Kolluri Poornima, Arul Prakash Francis, Muddasarul Hoda, Mohamed Ahmed Eladl, Srividya Subramanian, Vishnu Priya Veeraraghavan, Mohamed El-Sherbiny, Saad Mohamed Asseri, Abdulrahman Bashir Ahmed Hussamuldin, Krishna Mohan Surapaneni, Ullas Mony, Rukkumani Rajagopalan

**Affiliations:** ^1^ Department of Biochemistry and Molecular Biology, School of Life Sciences, Pondicherry University, Pondicherry, India; ^2^ Centre of Molecular Medicine and Diagnostics, Saveetha Dental College and Hospitals, Saveetha Institute of Medical and Technical Sciences, Saveetha University, Chennai, India; ^3^ Department of Biological Sciences, Aliah University, Kolkata, India; ^4^ Department of Basic Medical Sciences, College of Medicine, University of Sharjah, Sharjah, United Arab Emirates; ^5^ Department of Basic Medical Sciences, College of Medicine, AlMaarefa University, Riyadh, Saudi Arabia; ^6^ Department of Clinical Medical Sciences, College of Medicine, AlMaarefa University, Riyadh, Saudi Arabia; ^7^ Departments of Biochemistry, Molecular Virology, Research, Clinical Skills, and Simulation, Panimalar Medical College Hospital and Research Institute, Chennai, India

**Keywords:** 3D culture, biomimetic, microenvironment, cancer, gene expression, drug discovery

## Abstract

Replicating the naturalistic biomechanical milieu of cells is a primary requisite to uncover the fundamental life processes. The native milieu is significantly not replicated in the two-dimensional (2D) cell cultures. Alternatively, the current three-dimensional (3D) culture techniques can replicate the properties of extracellular matrix (ECM), though the recreation of the original microenvironment is challenging. The organization of cells in a 3D manner contributes to better insight about the tumorigenesis mechanism of the *in vitro* cancer models. Gene expression studies are susceptible to alterations in their microenvironment. Physiological interactions among neighboring cells also contribute to gene expression, which is highly replicable with minor modifications in 3D cultures. 3D cell culture provides a useful platform for identifying the biological characteristics of tumor cells, particularly in the drug sensitivity area of translational medicine. It promises to be a bridge between traditional 2D culture and animal experiments and is of great importance for further research in tumor biology. The new imaging technology and the implementation of standard protocols can address the barriers interfering with the live cell observation in a natural 3D physiological environment.

## Introduction

Since the 1940s, cells are consistently maintained by proliferation in specially designed culture media, adhered to glass or plastic surfaces (1). Hela cells are among the first cancer cells to be maintained and cultured consistently since 1951 (2). Such adherent-type cells proliferate in a specific culture medium and grow in a two-dimensional (2D) pattern. Such cell types may play a significant role in various cancer-based experiments; however, they have their share of limitations. The major hurdle in the 2D cell culture techniques is that they fail to replicate the three-dimensional (3D) microenvironment of biological tissues (3). The microenvironment includes the extracellular matrix (ECM) and neighboring cells, which are elementary for the formation and functioning of tissues and play a pivotal role in regulating tissue growth and development (4). The microenvironment is a connecting structure that explains cell behavior, identity, and function. It is not simply an arena to hold the cells in. The present 3D culture techniques can imitate the ECM elements, though the recreation of the exact microenvironment is still challenging (5).

Standard 2D cell cultures do not perform a satisfactory role in recreating a natural cellular environment (6). Hence, there is a demand for creating a reliable, controllable, and realistic culture systems that assist cell growth, differentiation, and organization resembling natural tissues and organs (7). Biomaterials like hydrogels have been developed to grow cells in three dimensions and to investigate countless cellular processes towards understanding morphogenesis, aging, and disease (8). 3D culture systems help in covering the gap between 2D monolayer cell cultures and the complexity of the organism (9). Cells growing in 3D cultures behave differently, and physiologically significant aspects can be studied more realistically. Those aspects include cell morphology, behavior, differentiation, proliferation rate, gene expression, and genomic profiles; malignant tumor formation, drug development, and testing; and *in vitro* culture of multi-cellular tissue for later implantation (10).

**Figure 1:**
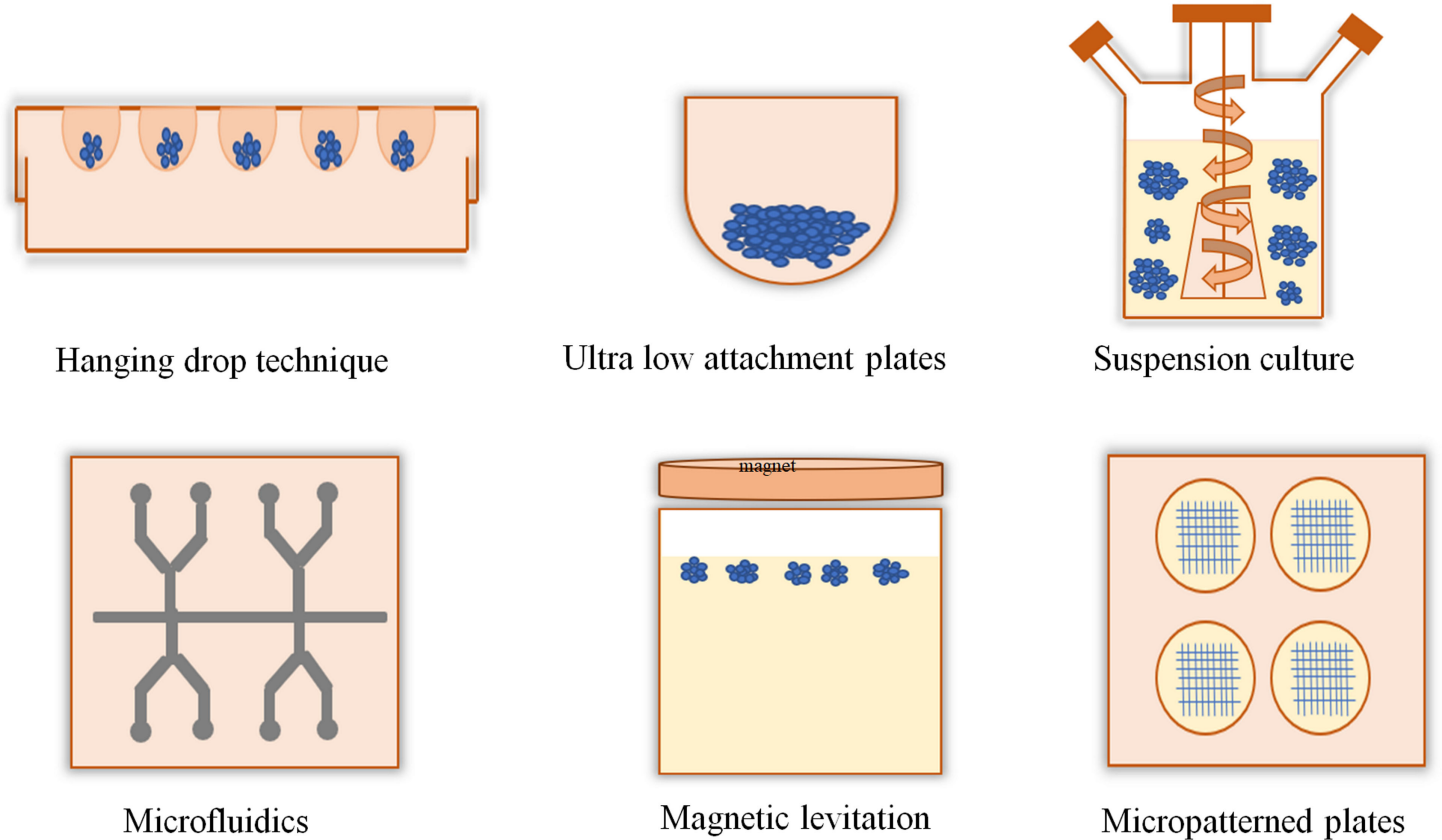
Scaffold-free and anchor-independent three-dimensional cell cultures. The hanging drop technique uses the surface tension of a droplet of cell suspension and gravity to suspend the droplet of cells onto the base of a lid which could promote cell aggregation into a spheroid. In the ultra-low attachment plate technique, the plate surface is coated with an inert substance that minimizes cell attachment and promotes cell aggregation. Suspension culture methods produce tumor spheroids using bioreactors, such as spinner flask and rotating flasks. Microfluidics can create uniform-sized spheroids for high-throughput screening applications. In the magnetic levitation method, cells with magnetic iron oxide were held at the air–medium interface using magnetic force. Micropatterning techniques control the position of cells and their shape.

**Figure 2:**
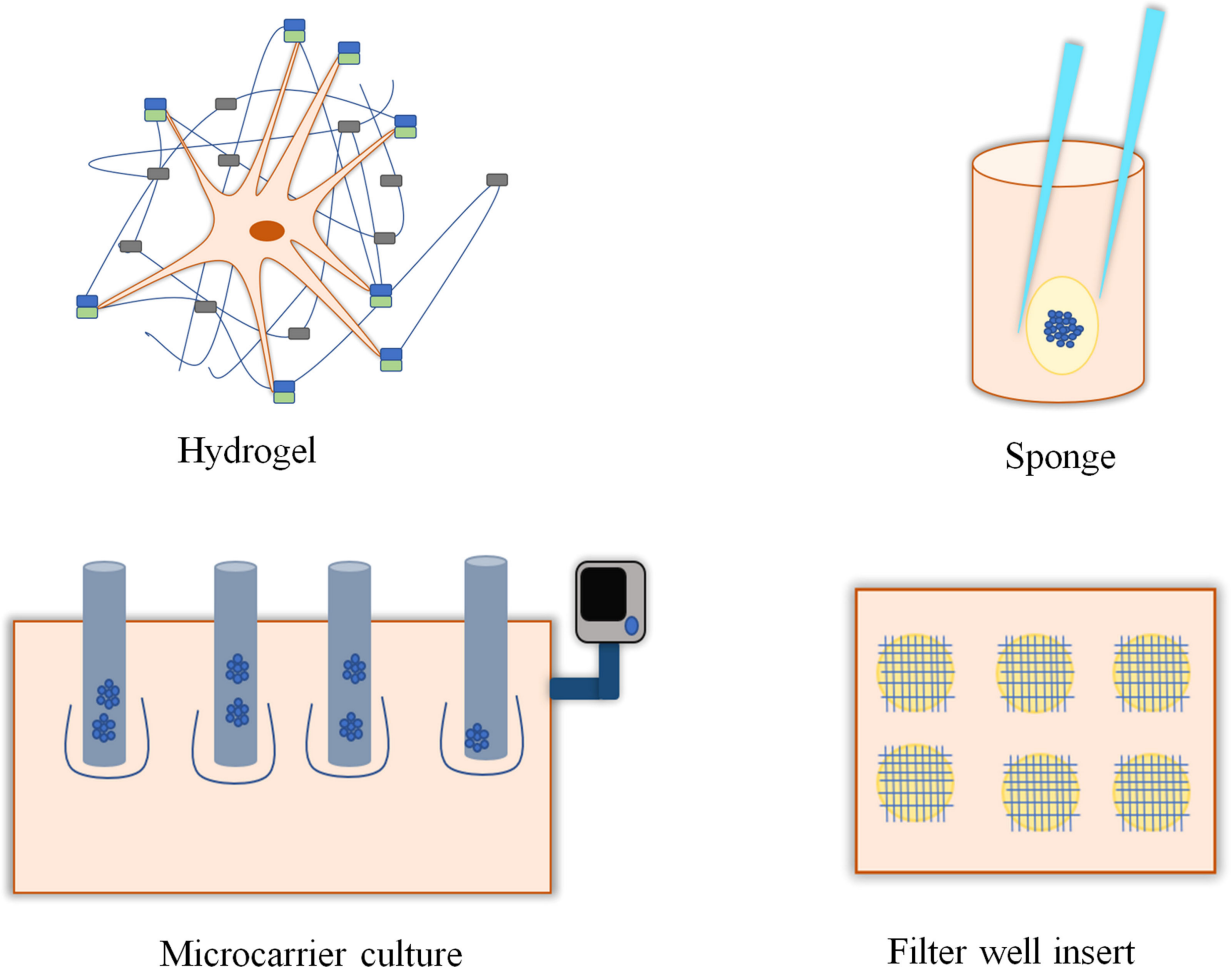
Scaffold-based and anchor-dependent three-dimensional cell cultures. Hydrogels with interconnected pores promote cell growth by providing a 3D environment and supporting the transport of oxygen, nutrients, and metabolites required for cell growth. Sponges, with their porous structure, support cell growth and migration. Microcarrier is a support matrix in bioreactors that supports the development of multi-cellular spheroids. Filter-well insert in culture plates promotes the growth of 3D cell culture.

The applications of 3D culture include tissue engineering or cell biology studies that emphasize the presence of cells in an artificial environment. Currently, there are no such models that can imitate the intricacy of the natural environment *in vivo*. In 2D cell culture, cells were grown only on the surface; on the other hand, in 3D culture, cells can migrate into the matrix and develop contacts in all dimensions (10, 11). The generation of 3D cell cultures may be broadly categorized depending on the presence of scaffolds. Techniques such as hanging drop, magnetic levitation, and ultralow attachments are typically classified as scaffold-independent 3D cell culture techniques (**Figure 1**), whereas 3D techniques including hydrogel, sponge, and microcarriers are categorized as scaffold-dependent 3D cell culture techniques (**Figure 2**). In many 3D cell cultures, the migration of cells in a confined 3D environment depends on microtubule dynamics, whereas in 2D, the migration depends on cycles of myosin-mediated contraction, integrin-mediated adhesion, and actin protrusion (12, 13).

## Major Research Challenges in 2D Cell Culture

The native 3D milieu of the cells is not sufficiently taken into account by 2D culture systems, *i*.*e*., cells in 2D culture appear stretched and flat compared to those in *in vivo* conditions (14). In traditional 2D cultures, cells are grown as a monolayer on flat plastic surfaces, allowing the cells to receive oxygen and nutrients from the medium throughout their growth in a homogenous manner. Proliferating cells form monolayers wherein the necrotic cells are easily removed while changing the culture medium. The abnormal morphology of cells in monolayers influences cellular differentiation, proliferation, gene expression, protein expression, and apoptosis (15). There is an increasing demand for *in vitro* models that can recapture the relevant complexity of *in vivo* milieu other than monolayer cultures (16, 17). Appropriate platforms of 3D culture act as outstanding *in vitro* models that allow the study of cell responses in a setting that mimics the native *in vivo* environments and curtail the need for animal trials, especially for toxicity assays (10). The physical and spatial aspects of the 3D platform influence the signal transduction between the cells, which, in turn, affects their behavior and gene expression (18). The 3D culture system offers spheroids, which have a higher ability to adhere and shield cells from harsh conditions. In spheroids, there is close mimicking of cell–ECM and cell–cell interactions that imitate the natural milieu found *in vivo* so that the morphology of the cells mimic the actual shape of cells in the tissues (19). Cells in a spheroid usually contain cells at various stages comprising necrotic, hypoxic, quiescent, apoptotic, and proliferating cells. The outer layer of the spheroid has viable and proliferating cells because it is more exposed to the medium. On moving to the core, cells receive fewer nutrients, growth factors, and oxygen from the medium and are present in a hypoxic or quiescent state (20). This cellular heterogeneity is a good replica of *in vivo* tissues, mainly in tumors. The cellular processes in 3D cultures are closely imitated *in vivo* because of the morphology and interaction of the cells (21).

The rate of proliferation of cells in 2D and 3D usually varies and depends on the matrix and cell line used. An array of endometrial cell lines (RL95-2, EN-1078D, KLE, and Ishikawa) display reduced proliferation rates when grown in 3D reconstituted basement membrane (rBM) compared to 2D culture and was identified with a drop in the proliferating cell nuclear antigen (PCNA) protein marker expression. The total number of cells in 3D rBM is decreased after 8 days of growth (22).

The differential expression of genes and proteins in a monolayer and 3D cultures is generally the speculation for the different behavior of mainly the genes which are involved in chemosensitivity, proliferation, migration, invasion, and angiogenesis (23). Loessner et al. (2010) (24) has reported that 3D ovarian cancer lines depict a significant increase in the mRNA expression levels of the receptors on the cell surface like β_1_, α_3_, α_5_ integrins, and protease—matrix metalloproteinase 9 when compared to cells in 2D culture. Monolayer culturing of cells is well adopted because it has helped in understanding the cell behavior; however, it is now established that 2D systems cannot recapture the complete depiction of the cells as present *in vivo*—for example, few vital cancer cell features like tumoral heterogeneity, genetic profile, and morphology are not accurately recaptured in monolayer cultures. The 3D cell culture models help to preserve the original shape, polarization, heterogeneity, and genetic profile of stromal and cancer cells (25).

## Importance of Extracellular Matrix

There is a growing interest in 3D cell culture after recognizing the importance of ECM in various cell functions and behavior. ECM affects the interaction of cells with other cells and their microenvironment, along with the spatial arrangement of cells in their milieu (26). ECM is not an irregular mixture of secreted components; it has instead a defined composition of biochemicals and typical geometrical structures triggering individual cellular responses like interaction and differentiation (27). The elemental composition of ECM includes water, polysaccharides, and proteins. ECM molecules consist of fibrous matrix proteins (*e*.*g*., elastin, collagens), glycoproteins (*e*.*g*., fibronectin), proteoglycans (*e*.*g*., syndecan, perlecan), glycosaminoglycans (*e*.*g*., hyaluronan, heparan sulfate), ECM-sequestered growth factors, *e*.*g*., hepatocyte growth factor, platelet-derived growth factor, transforming growth factor-β, vascular endothelial growth factor, and other secreted proteins such as protease inhibitors and proteolytic enzymes (28). Proteoglycans form a natural, hydrated, gel-like structure to fill the extracellular interstitial space. Proteoglycans perform various functions, including buffering, hydration, binding, and conferring force resistance properties (29). The composition of the ECM influences cell adhesion and migration (30). The sensitivity of ECM components to proteolytic enzymes decides the capacity of the cell to redesign the matrix to migrate through it. Basement membrane is secreted basically by epithelial and endothelial cells, which interrupts the transport of proteins and enhances mechanical stiffness (31). In the *in vivo* study model, cells are surrounded by ECM-associated signaling molecules and connective tissues consisting of mesenchymal cells, fibroblasts, and adipocytes. Every tissue has its own ECM with a particular topology and composition that is produced during tissue development (29, 32).

Natural ECM gels like fibrin and type I collagen are readily available and contribute to a number of physical and chemical cues which are required for inducing morphogenesis (33). The diverse cues which are present in natural ECM may become a limitation when trying to isolate an effect of a particular factor. In this case, many new synthetic biomaterials are being developed at a rapid pace and are used as 3D ECM to resemble the regulatory characteristics of natural ECM-bound growth factors for studying the basic biological processes. Synthetic gels are being tailored to imitate particular properties of ECM to provide a reproducible and well-controlled cellular niche. Synthetic ECM is particularly developed to trigger cellular adhesion through short peptide sequences that are recognized as integrin-binding domains within ECM proteins (34). Arginine–glycine–aspartic acid peptides derived from fibronectin are prototypes, and many other sequences which are recognized by several integrins and other adhesion receptors have been identified (35).

Luca et al. (2013) (36) have reported the precise analysis of how ECM can control the genotype and phenotype of routinely used colorectal cancer (CRC) cell lines like HT-29, CACO-2, LOVO, DLD-1, COLO-206F, and SW480 in a laminin-rich ECM 3D model. They studied the expression of EGFR, protein kinase B, and p42/44 mitogen-activated protein kinases (MAPKs) as EGFR stimulates the proliferation of cells *via* MAPKs. It is a well-established therapeutic target for the treatment of advanced CRC. The results suggest that there are alterations in the gene and protein expression levels of EFGR, phospho-AKT, and phospho-MAPK in 3D cells when compared to 2D culture. Kiss et al. (2013) (37) reported that interactions between cells and ECM in a matrigel culture can regulate cell morphology and upregulate the expression of chemokine receptors—CXCR7 and CXCR4—in prostate cancer cell lines like LNCaP, PC3, and DU145.

## Significance of 3D Cell Culture

3D cell cultures facilitate the study of challenges that are posed to an organism (4). Monolayer culturing triggers the cells to adopt to an artificial, rigid, and flat surface which may reshape the cell metabolism and reduce the functionality of cells. The results obtained from monolayer culture methods may not match the *in vivo* behavior of cells (1). In 3D culture, cells have interacted with ECM and neighboring cells in all dimensions, while in 2D cell culture, only a part of the cell surface is exposed to the adjacent cells in the monolayer. Several studies have demonstrated that 3D cellular morphological organizations have an intense impact on the morphogenesis, development, response to therapy, and gene expression profiles of the cell. Contrary to the monolayer models, 3D culture systems differentiate tumor phenotypes from non-malignant ones, where the tumor phenotype is embodied as multi-cellular organizations (37).

The contemporary 3D cell cultures, however, still lack the complex vascular systems that provide oxygen and nutrients and facilitate waste removal compared to *in vivo* systems. Cells grown in 3D cultures perform those functions by diffusion. Small spheroids do not face significant problems, but it becomes a challenge for large spheroids due to the lack of vasculature (38). Some reports suggest substantial progress in this direction (39). Expensive and highly complex culturing with complex downstreaming processes and an endpoint assay dependent on the culture method utilized are the various challenges of 3D cell culture (40). The advantages and disadvantages of different types of 3D culture techniques are summarized in **Table 1**.

**Table 1:** Brief comparison of various types of three-dimensional cell culture techniques.

Types of 3D culture	Advantages	Disadvantages	References
Hanging drop technique	Simple, consistent, and cost effective The shape and size of the spheroids are reproducible Specialized equipment is not required	Difficulty in changing the culture medium without disturbing spheroids	(41)
Agitation-based approaches	Changing the culture medium is relatively easy Large-scale production and long-term culture are possible	Cellular physiology can be modified due to the shear forces Varying spheroid sizes can hinder the spheroid selection for drug screening assay Do not have control over the spheroid size	(42)
Microfluidics	Multi-dimensional imaging capability makes it compatible for high-content screening (HCS) The cellular microenvironment can be controlled Convenient for high-throughput drug screening (HTS)	Requires special equipment for HTS Extensive characterization of the formed spheroids is difficult and expensive Post cell culture recovery is difficult.	(43)
Magnetic levitation	The spheroids grow faster than in any other commonly used methods There is no need for artificial extracellular matrix (ECM) as the spheroids form intrinsic ECM The size of spheroids is in range of square millimeters which better reproduces hypoxic and necrotic conditions found in tumors No specific medium is required for spheroids	The magnetic beads are expensive At high concentrations, magnetic beads are toxic to cells A limited number of cells can be produced	(44)
Low-attachment plates	Cost-effective when prepared in house Long-term culture and retrieval of cells are possible Enables HCS and HTS	The commercially available plates are expensive The spheroid size can be too heterogenous	(45)
Micropatterned plates	Simple and no extra equipment is required Have little well–well and plate–plate difference	Optimization utilizing the different patterns and adhesion properties infers control over 3D model location and geometry Bubbles can be formed easily in the media, which will disturb the culture	(45)
Inert matrix	The inert nature of the scaffold removes the issue of contamination arising from animal sources	Visualization under a light microscope is not possible Labor-intensive process	(46)
Hydrogels	Provide 3D support that almost mimics *in vivo* Incorporation of growth factors is possible	Expensive in case of large-scale production Limited commercial availability Advanced imaging technologies are required in some cases	(47)

## Role of 3D Cell Culture in Cancer Research

Cancer is the second major cause of death worldwide (48). Loss of tissue organization and the aberrant behavior of cellular components are characteristically observed in the pre-metastatic niche of cancer tissue (49). Early carcinogenesis stages include loss in cell polarity and detachment from the basement membrane, which allow the discrete accumulation of cells that communicate among them and with the surrounding microenvironment (50). Cancer cells reside in a complex microenvironment referred to as tumor microenvironment (TME), which contains various types of non-cancer cells and their stroma cells like endothelial cells, immune cells (lymphocytes and macrophages), fibroblasts, and mesenchymal cells. All these have a particular role in forming a specific structure, functioning, and physiology of the tumor (51).

The tumor and TME induce bidirectional changes in their functions and phenotypes that sustain the continuing process of tumor progression and metastasis under ephrin receptors and ligand interaction. These interactions evolve along with the progression of the disease (52). Hallmarks of cells in a tumor tissue include sustaining proliferative signaling, evading growth suppressors, resisting cell death, enabling replicative immortality, inducing angiogenesis, and activating invasion and metastasis (53).

In the last few decades, we have arrived at a better understanding of the genetic and molecular underpinnings of tumor etiology. However, concerns regarding the fast adaptation of drug resistance, dormancy, indolent disease, relapse, and metastatic colonization are inadequately answered by the classic monolayer culture models (54). Currently, the majority of cancer biologists rely on monolayer culture techniques to test the efficacy of experimental anti-tumor drug candidates before proceeding to *in vivo* testing because of their convenience (55). Conventional 2D platforms are well rooted and uncomplicated to use, but the lack of structural architecture and proper environment cause alterations in cell functions. When normal epithelial cells are grown in 2D monolayers, cells frequently lose the ability to differentiate, become highly plastic, and display characteristics exhibited by tumor cells. The malignant cells differ from their benign tumor counterparts (56). Tibbitt and Anseth (57) have illustrated that human breast epithelial cells grow like tumor cells when grown as monolayers, but when cultured in 3D fashion, they return to their natural growth behavior. A lack of a three-dimensional approach can generate uncertain experimental observations and produce inconsistent results. Furthermore, when cells grown in monolayer cultures are screened, they may negate the actions of some important compounds because of the adherence of the cells to plastic surfaces. Cells grown in monolayer culture are exposed to a homogenous environment with adequate oxygen and nutrients, whereas solid tumor cells are exposed to fluctuated critical biological and chemical signals, which can apply both inhibitory and stimulatory effects on tumor progression (58). Drug testing results may be affected due to the lack of 3D ECM network structures in 2D cultures. During anti-tumor drug testing, drugs applied to 2D cell culture can reach the cells without encountering any physical barriers, but when the same drug is delivered *in vivo*, they face a completely contrasting environment that originally reduces the drug’s partition throughout the complete tumor (59). Some vital cancer cell characteristics like morphology, tumoral heterogeneity, or genetic profile are not correctly imitated in monolayer cultures (11).

Researchers have developed 3D models on realizing the impediments of 2D cultures and the need for a complex native tissue microenvironment. 3D models can certainly recapitulate definite features of solid tumor tissue, like tumor morphology, multi-drug-resistant proteins and pro-angiogenic expression, varying distribution of biological and chemical factors, and reciprocal interactions between tumor and stroma (60).

The relentless development of tumor cell culture systems is crucial for studying tumor cell biology. In the last few decades, 3D cell culture technology has become an essential platform for basic research in cancer biology, utilizing an array of materials and processes to replicate the *in vivo* TME of cultured tumor cells (55). A number of studies highlight the organization of cells in 3D manner, revealing unique and unexpected visions into the tumorigenesis mechanism, and could show a valuable missing component in the *in vitro* cancer studies (61). Engineered 3D systems maintain a controllable and replicative microhabitat for incorporating ECM molecules, growth factors, specific cells, and other biochemical signals to better replicate the natural TME, favoring the growth and progression of tumor (59). **Table 2** summarizes various 3D cell culture models that have been designed and explored for cancer research.

**Table 2:** Three-dimensional cell culture models for cancer research experiments.

Type of 3D culture	*In vitro* model cell line	Type of cancer	Application (experimental study)	Outcome of the study	Reference
Hydrogel culture with collagen I	SH-SY5Y	Neuroblastoma	The growth response of human neuroblastoma cells in a 3D environment can be compared with those in a 2D environment	More than 1,700 genes were differentially regulated	(62)
Hydrogel culture with (GELFOAM) endosteal bone niche	MB-231, BoM1833	Breast cancer	Identification of genes regulating breast cancer dormancy in 3D bone endosteal niche	Only MB-231 showed dormancy; several dormancy-reactivation suppressor genes were identified	(63)
Hanging drop technique for spheroid formation Microfluidic bioreactor for proving *in vivo* TME	MDA-MD-231	Breast adenocarcinoma	Engineering a microfluidic bioreactor to examine the 3D breast tumor microenvironment	A robust microenvironment for studying the real-time migration of cancer cells along the matrix is fabricated	(64)
Hydrogel culture with Matrigel	Surgical specimens of pancreatic cancer patients	Pancreatic cancer	Develop and characterize patient-derived primary human pancreatic cancer organoids	Primary human organoids displayed a tumor-like cell morphology, tissue architecture, and polarity in contrast to cell line spheroids	(65)
Liquid overlay technique	PANC-1, MRC-5, HUVEC	Pancreatic adenocarcinoma	Conception and characterization of a novel 3D tumor model to mimic tumor complexity	A model combining the fibrotic tissue and a vessel-like structure, both hallmarks of pancreatic ductal adenocarcinoma, is constructed	(66)
Hydrogel culture with agarose	PC3 and DU145	Prostate cancer	Investigating the effect of the 3D arrangement on the expression of key epithelial to mesenchymal transition markers to better understand the prostate cancer cell behavior	Markers of the mesenchymal phenotype expressed at low levels	(67)
Hydrogel culture with puramatrix hydrogel	Specimens of bone marrow in premalignant or multiple myeloma (MM) conditions	Multiple myeloma	Construction of a 3D co-culture *ex-vivo* mesenchymal stem cell model to create multiple myeloma bone marrow niche	3D co-culture closely mimics the physiology of MM marrow	(68)
3D bioprinting (hydrogel)	BxPC-3, MIA, PaCa-2, and PANC-1	Pancreatic cancer	Evaluation of differences of the statin activity in 2D and 3D pancreatic cancer cell cultures	Statin-like pitavastatin demonstrated anti-cancer effects against selected pancreatic cancer cell lines	(69)
Matrigel	LOVO, COLO-205, CACO-2, COLO-206F, DLD-1, HT-29, SW-480	Colorectal cancer	To study the impact of 3D microenvironment on phenotype, gene expression, and EFGR inhibition of colorectal cancer cell lines	A specific spheroid growth pattern was observed in all investigated cell lines. DLC-1, HT-29, SW-480, and CACO-2 exhibited a clear solid tumor cell formation	(36)
3D bioprinting (hydrogel)	MDA-MB-231	Breast cancer bone metastasis	Hydrogel integration creates a biomimetic bone-specific environment suitable for breast cancer evaluation	3D matrix can mimic the tumor bone microenvironment, indicating that it can be used to study metastasis and assess drug sensitivity	(70)
Microfluidics (multi-organ chip)	NCI-H2N2, reconstructed human full-thickness skin	Lung cancer	Simultaneous evaluation of anti-EFGR-induced tumor and adverse skin effects in a microfluidic human 3D co-culture model	The combination of metastatic tumor environment with a miniaturized healthy organotypic human skin equivalent acts as an ideal tool for the evaluation of the therapeutic index of EFGR inhibitors	(71)
Bioreactor	Patient sample and A549	Lung cancer	Comparison of matrix metalloproteinases produced by human lung cancer cells in 2D and *ex vivo* 3D models	Human lung cancer cell grown in an *ex vivo* 3D lung model produces matrix metalloproteinases and is not produced in 2D culture	(72)
Hydrogel (collagen I and hyaluronan)	Patient sample	Lung cancer	Designing a 3D model to create lung cancer organoids	Lung cancer organoids exhibited anatomically relevant structures and lung cancer-specific behaviors	(73)
Microfluidics (Chip)	OSE, FTSEC, and OVCAR-8	Ovarian cancer	To devise a model to isolate exosomes from culture media and patient samples	Chip enables the isolation of exosomes and establishment of their protein profiles and associated signaling pathways in ovarian cancer	(74)
Hanging drop method	OVCAR3	Ovarian cancer	Designing an *in vitro* model to understand the chemoresistance and stemness in ovarian cancer	Late passage spheroids are significantly more tumorigenic with higher cancer stem cells than early passage spheroids	(75)

## Role of Microfluidics in Cancer Metastasis Research

Tumor metastasis is the migration of tumor cells from the primary site to colonize distant organs progressively. The progression of tumor metastasis takes place in a stepwise cascade manner, including tumor growth, angiogenesis, stromal invasion, intravasation, extravasation, and colonization at secondary sites within the body (76). The establishment and survival of metastases depend upon the microenvironment of specific receptive organs and the specific metastatic properties of the tumor cells inherited from the parental/primary tumor (77).

Metastasis is the major contributor to most cancer-related deaths. Treating cancer is a challenge because of biological heterogeneity and resistance of metastases to conventional drug therapy. Animal models have been instrumental in studying the cellular and molecular basis of the metastatic process. Due to the physiological difference between animals and humans, it has been problematic to determine the cause-and-effect relationships between specific biological cues and the resulting cancer cell behavior (76). Intermediate steps of the metastatic cascade cannot be satisfactorily studied using *in vivo* models. Hence, there is an immediate urge to develop technologies that directly control the physical and biochemical factors influencing tumor–vessel interactions (78).

Microfluidic platforms provide modeling systems for investigating various complex phenomena by combining an array of controllable biophysical and biochemical microenvironments with high-resolution real-time imaging. Bersini et al. devised a tri-culture microfluidic system with bone marrow-derived human mesenchymal cells lined with endothelium and MDA-MB-231 human breast cancer cell line to provide an osteo-conditioned environment. They have shown how breast cancer receptor CXCR2 and bone-secreted chemokine CXCL5 are involved in the breast cancer cell’s extravasation process. A profound understanding of the metastatic process can help develop therapeutic approaches that can increase the survival rate of cancer patients (79).

## Cancer-on-Chip

Cancer-on-Chip (CoC) is a microfluidic platform containing micrometer-sized compartments to millimeter-sized microchannels to facilitate controlled fluid transport. These compartments provide a space for recreating the niche where the mini-tumors can grow, develop, and interact with its specific microenvironment like *in vivo.* These chips contain chambers for cell culture where fluid flow, tissue mechanics, and composition of the microenvironment can be controlled. The present models of CoC help us to understand the role of the composition and structure of ECM in cancer cell invasion by using different imaging techniques to visualize the matrix (79). The successful development of a CoC model is based on the critical focus that mimics organ-level physiology or pathophysiology observed *in vivo*. Microfluidic models provide a significant advantage over static models, including transwells, spheroids, and organoid cultures (80). Wong and Searson (2014) (81) developed an artificial microvessel model which positions the tumor cells (dual-labeled MDA-MB-231 adenocarcinoma and HT-1080 fibrosarcoma cells) next to the artificial microvessel lined with endothelial cells [human umbilical vein endothelial cells (HUVEC) and adult human dermal microvascular endothelial cells] embedded in the ECM. They used live-cell fluorescence microscopy to inspect the interplay between the metastatic cancer cells and the endothelial cells in the artificial microvessel. They suggested that optimizing the pore size and stiffness in the ECM can enhance invasion by using low collagen concentration in dense matrices. Xie et al. (2020) (82) developed a unique 3D tumor array chip with a layer cake structure to screen the anti-cancer drugs epirubicin and paclitaxel. The chip contains the droplets of MDA-MB-231 breast cancer lines encapsulated with gelatin methacryloyl hydrogel. They observed that epirubicin could cause apoptosis at the core of the cell clusters at lower concentrations compared to paclitaxel. Based on this study, it was concluded that epirubicin was a more effective anti-cancer drug compared to paclitaxel (82).

A study on the co-culture of T47D human breast carcinoma cells with immortalized human mammary fibroblasts in fibronectin-rich ECM in a microfluidic device increased the growth of breast cancer cell clusters. This platform simplified the investigation of the morphology and growth of T47D cell clusters in response to broad-spectrum inhibitors of matrix metalloproteinases (83). A microfluidic model with a heterotypic co-culture approach using three different cell types (breast cancer cells, stromal cells, and monocytes) coupled with gene expression analysis exposed the interaction between different cell types through paracrine signaling *via* transforming growth factor-β production by breast cancer cells and corresponding receptor expression by stromal cells (84). Angiogenesis is a vital step in the cancer cascade as neovascularization is an essential control element restricting cancer progression and growth. Hence, researchers developed multiple microfluidic angiogenesis models that recreate capillary sprouting and vessel formation. The microfluidic angiogenesis model using primary human renal carcinoma cells produced a gradient of angiogenic stimuli that induced capillary outspread in co-cultured HUVECs (85, 86). A microfluidic device using ECM gel was developed, and it demonstrated the trans-endothelial cell migration of neutrophils *in situ* in response to chemical gradients (80, 87). A reconstituted glioblastoma tumor-on-a-chip model replicating the patient-specific resistances developed from concurrent chemoradiation and temozolomide treatment can be used to test different drug combinations with superior tumor killing potential (88).

CoC platforms have the potential to curtail the use of animal models as a complementary research tool. They can revolutionize the way of cancer research in terms of disease modeling, drug screening, and developing new therapeutic approaches specifically to prevent metastasis.

## 3D Cell Culture-Based Gene Expression Studies of Cancer

Gene expressions in the cells are susceptible to respond to even slight alterations in their microenvironment. Because of the physiological interactions with neighboring cells in 3D models, gene expression of cells is highly reproducible with minor modifications in 3D cultures (89). Spheroids of glioblastoma grown in 3D conditions are used in examining the different expressions of various genes, which play a vital role in tumor biosynthesis, metabolism, cell architecture, cellular transport, and signal transduction. It was observed that over-expression is analogous to *in vivo*, but not in the monolayer cultures. Kumar et al. (2008) (90) reported that when multiple genes of human neuroblastoma cells were screened through high-throughput screening methods, they revealed that the 3D matrix structural properties influence changes in gene expression. A microarray analysis of 1,766 genes featured the importance of neuroblastoma cells grown in 3D rather than as monolayers for studying diversity in gene expression (90). Similarly, Juillerat-Jeanneret et al. (2008) (91) reported a similarity in the heterogeneous gene expression of enzymes involved in detoxification and DNA repair, like O6-methylguanine-DNA-methyltransferase and glutathione-S-transferase, in 3D models of human glioblastoma cell line and *in vivo* (91). Such studies reveal that flexible 3D matrices should be optimized according to the application.

The advantage of gene expression measurements is that they provide insight into the biological mechanisms associated with the adaptation to a 3D environment (92). In *in vitro* studies, the adaptation of cells to a 3D environment is integral for maintaining transcriptional and translational functions. So that gene expression can match the *in vivo* level, Takahashi et al. (2015) (93) demonstrated that when hepatoma cell lines like HepG2 and Hepa RG were cultured using the hanging drop method, the expression of genes related to glucose, lipid, and drug metabolism was remarkably increased, the secretion of albumin and Apo B (Apolipoprotein B) was enhanced, and the mRNA levels of CYP (cytochrome P450) enzymes, on exposure to specific inducers, increased. While comparing the 2D and 3D culture models as drug-testing platforms in breast cancer, Imamura et al. (2015) (18) reported that the 3D model with the breast cancer cell line BT-474 had a lower level of caspase-3 than the 2D model, suggesting the anti-apoptotic nature of dense multi-cellular spheroid model of BT-474 cell line. Rodriguez et al. (2018) (94) reported that 3D architecture can modulate the breast cancer stem cell population and expression and distribution of HER2 receptors and create the environmental conditions to allow trastuzumab to exert its effect over the cells that are clinically defined as HER2-negative. Melissardou et al. (2019) (95) reported that when head and neck squamous cell carcinoma cell lines were cultured using ultra-low attachment plates, spheroids with varied sizes and densities were formed. All spheroids showed the upregulation of CDH1 (epithelial to mesenchymal transition-associated gene), NANOG, and SOX2 (stem cell markers) as compared to 2D cultures. This 3D model showed decreased sensitivity to cisplatin and cetuximab. When Fontoura et al. (2020) (96) compared the cell culture models of mouse melanoma (*in vivo* and cell line B16F10 GFP cells) using different scaffold-based techniques like electrospinning, solvent-casting particle-leaching, and Engelbreth–Holm swarm gel with 2D cultures, the microarray analysis of RNA showed the upregulation of lymphoid enhancer-binding factor 1 in 3D models than in 2D.

## Role of 3D Cultures in Cancer Drug Discovery and Development

Drug discovery and development is a complex and lengthy process and generally involves the same trend of progression (97, 98). Cell-based assays have become an integral part of the drug discovery process, facilitating a fast, simple, and cost-effective tool and thereby minimizing cost-intensive and large-scale animal testing (28). Lack of efficacy is the main reason for the failure of about 65% of drugs in phase II and phase III clinical trials. About one-third of drugs fail due to safety issues and inadequate therapeutic index (99). Although conventional *in vitro* human cell culture platforms are economical and easy for screening experimental targets, they cannot imitate the complex and dynamic responses of human organs. Phenotype recreation of the target tissue in cultured cells is crucial for procuring reliable bio-medical data. Drug discovery and development researchers are interested in 3D culture systems because of their advantages in contributing to more anticipating and physiologically consistent data for *in vivo* tests (11). 3D cultures assist the initial drug discovery process starting from disease modeling for identifying the target and its validation, screening, lead selection, analysis of efficiency, and safety assessment (100). Among the various criteria for drug development, hepatotoxicity is an essential determining parameter for the approval, non-approval, or limitation in drug usage by the Food and Drug Association (101). Utilization of hepatocarcinoma cell line (HepG2) grown as monolayers has been the gold standard in the initial screening of drug candidates for hepatotoxicity activity; however, the spheroid culture of hepatocytes is fast replacing the 2D culture (102). Furthermore, the cells in this setup either wholly lack or express drastically low levels of transporters and many cytochrome P450s (CYPs), which are drug-metabolizing enzymes found in hepatocytes *in vivo* (103). When hepatocyte cultures are accomplished in bioreactors, they have been shown to imitate *in vivo* characteristics to such an extent that they can be utilized for the bio-fabrication of a functional artificial organ for transplantation (103). The structure and function of the mammary gland and/or steps involved in breast cancer progression have been studied in the *in vitro* 3D scaffolds that imitate the same. Silk fibroin hydrogels with good biocompatibility and processability can be engineered further to encourage normal tissue regeneration or to enhance the tumorigenicity of cancer cells.

On the other hand, decellularized ECM plays a significant role as a novel tissue-specific ECM material that matches the native tissue matrix (104, 105). Yi et al. (2016) developed a 3D printed polymeric patch loaded with 5-fluorouracil using an extrusion-based printing system made of PCL and poly(lactide-coglycolide). This resulted in sustained drug release and significantly reduced pancreatic tumor in a rabbit model (106).

Cells in 3D culture typically display decreased sensitivity to chemotherapeutic agents when compared to cells in 2D culture (107). The benefits of using 3D cultures in drug discovery include cell–cell contact, communication, and signaling pathway activation due to the ECM components of matrices. In addition, 3D culture helps in the morphological and functional differentiation of cells (108). 3D culture contributes to *in vitro* models for building multi-cellular systems. It covers the divergence between *in vitro* and *in vivo* drug screening, possibly reducing animal trials.

## Limitations and Challenges of 3D Cell Culture

In spite of the continued effort and considerable success in making biomimetic 3D tumor models, major challenges and impediments still accompany the existing models. Several 3D models are generated using the longstanding 2D adapted cancer cell lines, which might not exactly replicate the pathology of the disease (109). *Ex vivo* culturing of patient cells provides access to a high level of accuracy in finding the biological insights of metastasis and recognizing the susceptibility to drugs targeting the pathways in a correct manner (110). The remodeling of the complex vascular system associated with tumor *in vitro* is another important challenge necessary to be addressed because cancer progression is influenced by the abnormal blood vessels and greatly affect drug transport within the tumor tissues. A quantitative understanding of the movement of tumor cell aggregates in a cancer microenvironment is needed. Cancer cells are generally trapped at a single-cell state, proliferate within the matrix, and simultaneously migrate through mesenchymal or amoeboid movement, which is vital for forming tumor cell aggregates (111). Cancer biologists and tissue engineers are required to team up to create an optimized 3D *in vitro* model of drug testing and to make progress in this field. Systems biology, computational modeling approaches, detection and analysis modalities, and *in situ* real-time imaging, are severely required for a better understanding of 3D tumor models. These advancements are expected to lead to better insights into the largely illusory aspects of cancers.

Differences in the biologically derived matrices might generate non-reproducible results. High-throughput assays and large-scale studies are expensive. Within the same flask, spheroids of varying sizes may be produced. 3D models do not contain vasculature, which is important in drug delivery. The three-dimensional cultures must benefit the drug discovery and clinical research as it generates physiologically relevant screening assays (112). The national institutes of health, hospitals, and university laboratories are shifting their emphasis on identifying culture models with clinical potential.

## Conclusion

Cell culture has been the elemental tool to unveil the biochemical mechanisms underlying the assembly of cells into tissues, organs and, finally, an organism. Conventional cell culture models have played a significant role in achieving so, but they fail to exactly translate the natural *in vivo* setting. The process of carcinogenesis is not revealed entirely because implementing the culture techniques could not recreate the milieu of the tumor and its TME. 3D cultures can explore new ways to understand the biological processes of various diseases, mainly cancer. Bioengineered 3D tumor models have the potential to reveal the whole scenery of cancer and can help in developing new therapeutic approaches.

## Author Contributions

Conceptualization: APF and RR. Writing—original draft preparation: KP, SS, and MH. Writing—review and editing: MH, APF, and RR. Resources: VPV, UM, and KMS. Funding acquisition: MAE, ME-S, SMA, and ABAH. All authors contributed to the article and approved the submitted version.

## Funding

This publication was supported by AlMaarefa University Researchers Supporting Program (grant number: MA-006), AlMaarefa University, Riyadh, Saudi Arabia.

## Conflict of Interest

The authors declare that the research was conducted in the absence of any commercial or financial relationships that could be construed as a potential conflict of interest.

## Publisher’s Note

All claims expressed in this article are solely those of the authors and do not necessarily represent those of their affiliated organizations, or those of the publisher, the editors and the reviewers. Any product that may be evaluated in this article, or claim that may be made by its manufacturer, is not guaranteed or endorsed by the publisher.
